# What is positive youth development and how might it reduce substance use and violence? A systematic review and synthesis of theoretical literature

**DOI:** 10.1186/s12889-016-2817-3

**Published:** 2016-02-10

**Authors:** Chris Bonell, Kate Hinds, Kelly Dickson, James Thomas, Adam Fletcher, Simon Murphy, G. J. Melendez-Torres, Carys Bonell, Rona Campbell

**Affiliations:** 1 Department of Childhood, Families and Health, University College London Institute of Education, 18 Woburn Square, WC1H 0NR London, UK; 2 Cardiff School of Social Sciences, Cardiff University, CF10 3BD Cardiff, UK; 3 Division of Health Sciences, Warwick Medical School, University of Warwick, CV4 7AL Coventry, UK; 4 Forest School, 2 College Place, E17 3PY London, UK; 5 School of Social and Community Medicine, University of Bristol, Canynge Hall, 39 Whatley Road, BS8 2PS Bristol, UK

**Keywords:** Theory of change, Positive youth development, Smoking, Alcohol, Drugs, Violence

## Abstract

**Background:**

Preventing adolescent substance use and youth violence are public health priorities. Positive youth development interventions are widely deployed often with the aim of preventing both. However, the theorised mechanisms by which PYD is intended to reduce substance use and violence are not clear and existing evaluated interventions are under-theorised. Using innovative methods, we systematically searched for and synthesised published theoretical literature describing what is meant by positive youth development and how it might reduce substance use and violence, as part of a broader systematic review examining process and outcomes of PYD interventions.

**Methods:**

We searched 19 electronic databases, review topic websites, and contacted experts between October 2013 and January 2014. We included studies written in English, published since 1985 that reported a theory of change for positive youth development focused on prevention of smoking, alcohol consumption, drug use or violence in out-of-school settings. Studies were independently coded and quality-assessed by two reviewers.

**Results:**

We identified 16 studies that met our inclusion criteria. Our synthesis suggests that positive youth development aims to provide youth with affective relationships and diverse experiences which enable their development of intentional self-regulation and multiple positive assets. These in turn buffer against or compensate for involvement in substance use and violence. Existing literature is not clear on how intentional self-regulation is developed and which specific positive assets buffer against substance use or violence.

**Conclusions:**

Our synthesis provides: an example of a rigorous systematic synthesis of theory literature innovatively applying methods of qualitative synthesis to theoretical literature; a clearer understanding of how PYD might reduce substance use and violence to inform future interventions and empirical evaluations.

**Electronic supplementary material:**

The online version of this article (doi:10.1186/s12889-016-2817-3) contains supplementary material, which is available to authorized users.

## Background

Adolescent use of tobacco, alcohol and drugs (henceforth termed substance use) is an important threat to public health leading to later-life chronic disease [1, 2]. Surveys suggest that a fifth of US adolescents nearing the end of high school engaged in binge drinking in the last month [3]. Around a quarter of US adolescents in the second year of high school report illicit drug use in the last year [4]. Use of substances such as tobacco and illicit drugs is subject to social determinants acting at the individual, peer, family and community level, with important implications for health inequalities [5]. Preventing youth violence is another public health priority [6–8]. A quarter of UK youth age 15–16 years have carried a weapon and a fifth report attacking someone with intent to hurt them seriously [9]. Violence is subject to marked inequalities [10] and is associated with increased risk of: physical health problems; [11] engaging in other health risk behaviours such as substance use; [12–14] long-term emotional, behavioural and mental health problems; [11, 15, 16] and self-harm and suicide [17]. The economic costs associated with youth substance use and aggression are extremely high [18–20].

There are increasing calls for youth interventions to address multiple risk behaviours since such behaviours cluster together, [21, 22] and because combined interventions are potentially more efficient [23]. Positive youth development (PYD) is one such intervention. In the UK, PYD has been defined as voluntary educational activities aiming to promote generalised positive development, in terms of skills, attitudes, relationships and identities, rather than merely preventing problem behaviours [24]. In the USA, PYD has been defined as voluntary education outside school hours aiming to promote generalised (not just health) and positive (not just avoiding risk) development of assets such as bonding, resilience, social, emotional, cognitive, behaviour or moral competence, self-determination, spirituality, self-efficacy, clear and positive identity, belief in the future, recognition for positive behaviour, opportunities for pro-social involvement and/or pro-social norms [24, 25]. Interventions are said to qualify as PYD if they address multiple assets or a single asset applied to multiple domains such as the family or local community [25].

PYD interventions are widely deployed, [26] often with the aim of preventing substance use and violence [27, 28]. Existing reviews suggest PYD can reduce violence and drug use albeit with considerable heterogeneity of effects, [29, 30] but these reviews vary in how systematic they are, and are becoming out of date. A more recent review focused only on school extra-curricular interventions reported a significant effect reducing problem behaviours but not drug use [31]. Each of these reviews focused exclusively on empirical evidence for intervention effectiveness.

However, empirical studies of the effectiveness of particular interventions are insufficient to tell us whether the PYD approach is effective unless we are clear what theory of change this approach involves and whether particular interventions embody it. What is needed is a theory of change defining what PYD interventions involve and the intended causal mechanisms via which they are intended to reduce substance use and violence. This would help determine whether existing intervention studies provide evidence about the effectiveness of the PYD approach or not. It could also inform the design of future PYD intervention studies so that they provide evidence about the effectiveness not only of the specific intervention in question but on the overall PYD approach. However, no existing systematic review has synthesised PYD theories of change. The synthesis of empirical evaluations of PYD interventions also performed as part of this overall review to be published shortly found that included empirical evaluations assessed under-theorised interventions and so provide little guidance on the effectiveness or otherwise of the PYD approach. A synthesis of the theoretical literature could thus make an important contribution to improving the quality of PYD interventions being evaluated and thus of the evidence base for this approach.

Therefore, as part of a broad review also examining empirical evidence, we undertook a systematic review to examine theoretical literature on the PYD approach to preventing substance use and violence. As mentioned above, our synthesis of the empirical evidence will be published in due course.

Increasing interest in theory synthesis reflects growing recognition of the importance of understanding mechanisms in intervention research [27, 28, 32]. Theory synthesis differs from meta-theory in aiming to compare and integrate closely related theories [28]. This best describes our own goal in synthesising a relatively cohesive PYD theoretical literature united by its use of the term ‘positive youth development’ and focus on how such interventions might reduce violence and substance use. Our aims were first to develop a clearer normative understanding of what is meant by PYD in terms of its goals. Second, we aimed to develop a comprehensive causal theory of change for how PYD might prevent substance use and violence.

## Methods

Methods were determined a priori, described in a protocol, [33] and followed PRISMA guidance [34] (See Additional file 1. Studies were included in the overall review if they: were published since 1985; were in English; focused on youth age 11–18 years; focused on PYD as defined in our introduction; reported a theory of change, process evaluation or experimental/quasi-experimental outcome evaluation; and focused on prevention of smoking, alcohol consumption, drug use or violence in out-of-school settings. Reports included in the theory synthesis were required to describe what PYD involves and how it is intended to reduce substance use or violence.

We searched 19 bibliographic databases, including PsycINFO, MEDLINE and ERIC, plus topic-specific websites, trials registers and experts between October 2013 and January 2014. For a complete list of sources and approach to searching refer to the published protocol [33]. http://www.crd.york.ac.uk/prospero/display_record.asp?ID=CRD42013005439). We used indexed and free-text terms related to population (for example, youth) AND intervention (for example, informal education) OR population/intervention (for example, youth work). An example is provided in Additional file 2. References were initially screened on title/abstract then full report if the title or abstract suggested relevance or provided insufficient information to judge. At both stages, screening was initially done by pairs of researchers assessing batches of the same 100 references, moving to single screening once 90 % agreement was achieved.

We extracted data on: aim; description of theory; and links to other theories. Data-extraction tools were piloted on two studies with reviewers meeting to identify differences and refinements. All reports were then extracted by two reviewers who then discussed and agreed coding. Unlike most theory syntheses, [35–37] we aimed to assess study quality and use this to determine which studies were given most weight in our synthesis. Study quality was assessed by pairs of reviewers (from CB, KH, JT) independently, using a new tool informed by a previous review [27] and methodological work [38, 39]. Quality was assessed in terms of: construct clarity; clarity of relationships between constructs; empirical testability; parsimony; and potential generalisability. Reviewers were provided with guidance to inform decisions. Reviewers settled differences through discussion.

We aimed to synthesise theoretical literature. To do so, we innovatively applied methods previously applied to synthesising qualitative research [40]. We used a form of qualitative analysis known as template analysis [41]. Reviewers first developed an a priori template which included PYD theoretical constructs already known to us (Table 1). The two reviewers independently used this template to code two theory papers (chosen on the basis that both reviewers had assessed these as high quality), refining the theoretical constructs within template in the light of their reading of these papers and writing ‘memos’ to explain their rationales for doing so. The reviewers then discussed their refined templates, developing an agreed version. The two reviewers then coded the remaining theory papers, further refining the coding template as they went along and drafting an overall memo explaining their refinements and summarising their emerging overall synthesis. Reviewers kept a record of the coding template and their overall memo as it stood at the end of coding each paper. At the end, the reviewers compared and combined their memos to produce an overall summary of their analysis.

**Table 1:** Initial coding template

Themes	Codes and sub codes
*Definition of PYD interventions*	PYD versus prevention science/traditional youth programmes
	Definition in terms of developmental assets versus programme atmosphere
	Characteristics of programmes
*Taxonomy*	Individual versus environment/community emphasis
	Pro-social development versus critical conscious raising
	General ‘pile up’ of assets versus specific ‘molecular’ effects of specific assets on specific outcomes
*Mechanism of action*	Action on risk of SU/violence
	Action on thriving
*Possible moderation by context*	Moderation by person/population
	Moderation by setting

## Results

**Fig. 1:**
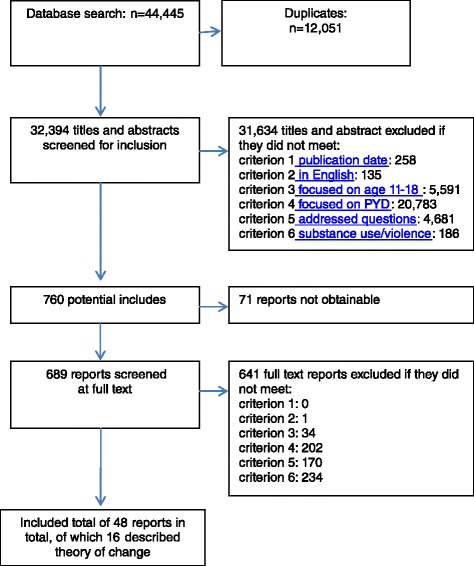
Screening

The searches provided 32,394 unique references, of which 16 reports [30, 42–56] met the inclusion criteria for the theory synthesis (see Fig. 1), described in Table 2. One report originated from Canada [47] and one from Hong Kong [52] while the remainder were from the USA [30, 42–46, 48–51, 53–56]. One also reported a process and outcome evaluation [46]. Most referred to established theories, such as ecological systems theory, [42] social learning theory, [46] and identity development theory [47] in setting out a theoretical basis for PYD preventing substance use and violence. Although all studies described what is meant by PYD and offered some insights into how PYD might reduce violence or substance use, only nine studies addressed mechanisms in depth [30, 44, 46, 48, 50–52, 55, 56].

**Table 2:** Characteristics of theory reports

Author and title	Stated aims	Existing theories cited
Benson et al. [19] Youth development, developmental assets, and public policy	1. Describes the strength-based youth development approach by comparing it to and contrasting it with the deficit-based orientation to successful development. 2. Discusses the theoretical and empirical basis of the developmental asset framework as a prime exemplar of positive youth development, a comprehensive conceptualization of developmental well-being, and a generator of knowledge regarding the developmental pathways of young people. 3. Identifies relevant social and cultural dynamics affecting youth, considers their implication for youth development policy, and highlights a number of public policies from around the country that reflect the tenets and unfolding wisdom of healthy youth development. 4. Assessing the socio-political prospects for developmental principles and knowledge to actually inform and shape public policy for young people.	Ecological model of human development
Benson [18] Developmental assets: an overview of theory, research and practice	1. Discusses the concepts of developmental assets, asset building communities and asset building society. 2. Discusses the 4-H survey in relation to assets and damaging behaviours.	Developmental systems theory Action theory of development Ecological model of human development
Benson [20] Positive youth development and the prevention of youth aggression and violence	1. Reports analyses on several databases of 6th-12th grade students in the United States, to explore the linkage of positive relationships, opportunities, skills, and values, called Developmental Assets, to prevention of youth aggressive and violent behaviours.	Ecological Theory
Benson et al. [21] The contribution of the developmental assets framework to positive youth development theory and practice	1. Synthesises literature on developmental assets. 2. Discusses the recent development of: the Developmental Asset Profile, an instrument designed, in part, to assess change-over-time; the utilization of asset measures in international research; the expansion of the assets framework to early childhood and young adults; and new research using latent class analysis (LCA) to identify classes or subgroups of youth.	-
Berg et al. [22] Youth Action Research for Prevention: a multi-level intervention designed to increase efficacy and empowerment among urban youth	1. Reports on the theory of change for and empirical evaluation of the Youth Action Research for Prevention program.	Ecological systems theory Identity theory Social learning theory Social construction theory Critical Transformational Theories
Busseri et al. [23] Breadth and intensity: salient, separable, and developmentally significant dimensions of structured youth activity involvement	1. Presents a theory-based framework for studying structured activity involvement (SAI) as a context for positive youth development based on two key dimensions: breadth and intensity of involvement. 2. Demonstrates the separatability, salience, and developmental significance of these two dimensions.	Identity development theory Life-span development processes of selective optimization with compensation Concept of ‘affordances’ in *Gibson’s* ecological theory of human perception
Catalano et al. [24] Prevention science and positive youth development: competitive or cooperative frameworks?	1. Examines the convergence in the critiques and recommendations for the future of programs to promote healthy development and prevent problem behaviors among children and adolescents.	Attachment theory Identity development theory Ecological model of human development
Ginwright and Cammarota [25]New Terrain in Youth Development: The Promise of a Social Justice Approach.	1. Presents a youth development model that addresses structures of power and teaches youth to understand how their opportunities are circumscribed by larger political, economic, and social forces. 2. Critiques two dominant approaches to youth development which have oppressed urban youth of colour.	Critical consciousness theory
Kia-Keating et al. [26] Protecting and promoting: an integrative conceptual model for healthy development of adolescents	1. Draws on extant research to delineate linkages between the risk and resilience and positive youth development literatures.	-
Kim et al. [27] Toward a new paradigm in substance abuse and other problem behavior prevention for youth: youth development and empowerment approach	1. Addresses a paradigm shift taking place in the field of substance abuse prevention directed for youth. 2. Introduces an innovative approach to substance abuse and other problem behaviour prevention that reflects this shift in prevention paradigm.	Social control theory Social learning theory Social development model Problem behavior theory Expectations-states theory
Lee [28] Construction of an integrated positive youth development conceptual framework for the prevention of the use of psychotropic drugs among adolescents	1. Constructs an integrated conceptual framework for the prevention of adolescents’ use and abuse of psychotropic drugs. 2. Provides empirical support for integrating a positive youth development perspective in the revised model.	Social learning theory Symbolic interaction theory Operant conditioning theory
Lerner and Lerner [29] Toward a New Vision and Vocabulary About Adolescence: Theoretical, Empirical, and Applied Bases of a ‘Positive Youth Development’ Perspective	Sets out a new vision and vocabulary about adolescence in terms of theoretical, empirical, and applied bases of a ‘positive youth development’ perspective.	Developmental systems theory
Lerner et al. [30] Individual and contextual bases of thriving in adolescence: a view of the issues	1. Describes the relational developmental systems theory-based, positive youth development (PYD) perspective that frames much of contemporary research about health and positive development across the adolescent period and that, more specifically, frames the 4-H Study of PYD.	Bioecological theory Action theory models of intentional, goal-directed behaviours Life-course theory Dynamic systems theory Holistic person-context interaction theory Developmental systems formulations
Perkins et al. [31] Community Youth Development: A Partnership for Action	1. Introduces the concept of Community Youth Development.	-
Roth and Brooks-Gunn [12] Youth development programs: risk, prevention and policy	1. Focuses on the promise and reality of youth development programs. 2. Reviews the available evidence about program effectiveness. 3. Defines the elements of youth development programs based on theoretical writings and ethnographic studies. 4. Investigates the reality in two ways, by mapping the defining principles of youth development to practice by looking at which elements are present in successful programs, and by investigating the relation between these elements and program outcomes.	-
Schwartz et al. [32] Addressing the challenges and opportunities for today’s youth: toward an integrative model and its implications for research and intervention.	1. Calls for, and proposes some tenets of, model building in adolescent psychosocial development. 2. Suggests that there is a need for a model that draws from the risk-protection approach, from which many prevention science approaches are drawn, and the applied developmental science perspective, from which many positive youth development approaches are drawn.	Selection, Optimization and Compensation Model Theory of planned behaviour

The two reviewers produced independent quality scores for each report but found the criteria difficult to apply (Table 3) and could agree a common score for only three studies. We therefore decided to include theory reports in our synthesis regardless of quality.

**Table 3:** Quality assessment of theory studies

Paper	Clarity of constructs		Clarity of relationship between constructs		Testability		Parsimony		Generalisability		Total score	
	*CB/JT*	*KH*	*CB/JT*	*KH*	*CB/JT*	*KH*	*CB/JT*	*KH*	*CB/JT*	*KH*	*CB/JT*	*KH*
Benson et al. [19]	0	0.5	0	0.5	0	0	0	0	1	1	1	2
Benson [18]	0	0.5	1	0	1	0	0	0	1	0	3	0.5
Benson et al. [20]	1	0	0.5	0	1	0	0	0	1	1	3.5	1
Benson et al. [21]	0	1	0	0.5	1	1	0	0	1	1	2	3.5
Berg et al. [22]	0	1	0	1	0	1	0	1	1	1	1	5
Busseri et al. [23]	1	1	0.5	1	1	1	1	1	1	1	4.5	5
Catalano et al. [24]	0.5	0.5	0.5	0.5	1	0	1	0	1	1	4	2
Ginwright and Cammarota [25]	0	0	1	0	0	0	1	0	1	0	3	0
Kia-Keating et al. [26]	0	1	0	1	1	0	0	1	1	1	2	4
Kim et al. [27]	1	1	1	1	1	1	1	1	1	1	5	5
Lee [28]	0	0	0	0.5	0	0	0	0	1	1	1	1.5
Lerner and Lerner [29]	1	0.5	0.5	1	1	1	1	1	1	1	4.5	4.5
Lerner et al. [30]	1	0	1	0.5	1	0	1	0	1	1	5	1.5
Perkins et al. [31]	1	0	1	0	0	0	0	0	1	1	3	1
Roth and Brooks-Gunn [12]	1	1	1	0	0	0	1	1	1	1	4	3
Schwartz et al. [32]	1	0	1	0	1	0	1	0	1	0	5	0
These scores were agreed between KH and JT												
Ginwright and Cammarota [25]	0		0		0		1		1		2	
Perkins et al. [31]	1		1		0		0		1		3	
Roth and Brooks-Gunn [12]	1		1		0		1		1		4	

Key: 1 = Yes, 0.5 = Partial, 0 = No

**Fig. 2:**
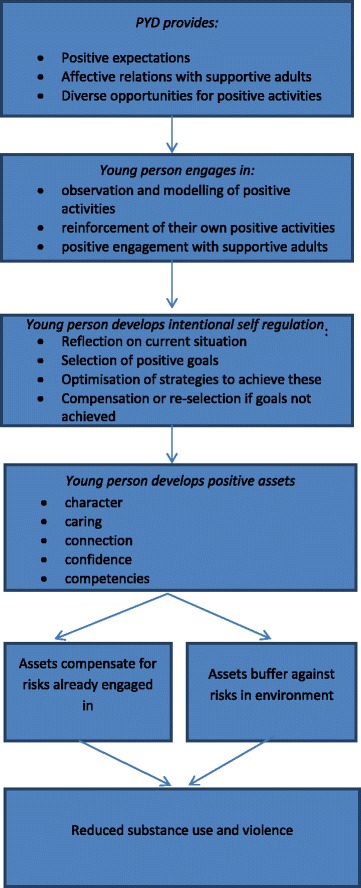
Synthesised theory of change for PYD effects on substance use and violence

Despite variation in what aspects of the potential causal pathway studies examined, these did not contradict each other and were sufficiently complementary to enable an overall synthesis to be developed. The syntheses produced by each reviewer differed. Both reviewers judged that many included reports did not on their own describe a comprehensive theory of change for how PYD might reduce substance use and violence, some for example focusing on particular sub-sections of the causal chain. One reviewer concentrated her synthesis on normative theory about PYD goals. The other developed a synthesis of normative and causal theory, finding that the normative theory was helpful in piecing together a rather fragmentary literature to develop a more comprehensive theory of change (Fig. 2). Because two reviewers synthesised in parallel, these differences in approach were transparent and could be used to provide depth and breadth to the review. The following themes were apparent:

### Normative theory of PYD

A major theme in the literature was what are the underlying goals of PYD programmes. These focused on the need to increase young people’s assets and ability to thrive, by developing affective relationship with providers and engagement in a diverse range of activities offered by PYD programmes.

#### Thriving and positive assets

The major emphasis across nearly all the literature was on the importance of enabling young people not merely to avoid risk behaviours but to achieve “*normal development*” (Roth and Brooks-Gunn [30] p.172). Kim et al. [51], Catalano et al. [48], Busseri et al. [47] and Roth and Brooks-Gunn [30] for example all state that “*problem free is not fully prepared*” (Roth and Brooks-Gunn [30] p.170). Lerner and Lerner [53] and Kim et al. [51] all contrast PYD with prevention science, arguing that PYD aims to develop various positive assets rather than simply focusing on preventing risk behaviours. PYD is described as a “*strength-based approach*” (p.781):

“*The theory and research undergirding developmental assets … are designed, in part, to reframe the targets and pathways of human development around images of strength and potential. We posit that this shift is crucial for mobilizing both personal and collective efficacy on behalf of child and adolescent development.*”

Roth and Brooks-Gunn [30], Busseri et al. [47], Lerner et al. [53, 54], Schwartz et al. [56] and Perkins et al. [55] all build on this idea of thriving by suggesting what particular assets PYD might aim to develop, terming these the “5 Competences”:

*“(1) competence in academics, social, emotional, and vocational areas;*

*(2) confidence in who one is becoming (identity);*

*(3) connection to self and others;*

*(4) character that comes from positive values, integrity, and a strong sense of morals;*

*(5) caring and compassion” (*Perkins et al. [55] p.50).

Benson et al. [42, 43, 45] and Roth and Brooks-Gunn [30] propose an alternative categorisation of 40 assets which, though similar to the above list, divide up between internal assets possessed by individuals and external assets possessed of environments which should nurture positive development.

#### Affective relationships with adults

Several authors, such as Roth and Brooks-Gunn [30], Benson and Scales, [44] Lerner and Lerner [53] and Kim et al. [51] argue that PYD interventions should involve providers developing affective (sustained, supportive and emotionally expressive) relationships with young people. They contrast these with provider-client relationships in conventional youth services which tend to be more narrowly instrumental (focused on providing specific services such as education or careers advice). It is argued that PYD providers should create a “*family-like atmosphere*” [30] (p.172) characterised by enduring and emotionally engaged relationships.

#### Diverse activities and settings

Many of the included reports assert that PYD interventions should offer diverse activities and settings for participants. Lerner and Lerner [53] and Benson et al. suggest that such diversity enables young people to develop broad skills. Such activities provide multiple opportunities for recognition [30]. Benson et al. suggest that PYD activities should involve “*synergies*” and “*redundancies*” (p.210). In other words, PYD interventions should provide young people with opportunities to develop the same assets in the course of different activities, so that these mutually reinforce each other.

### Causal theory of PYD

Another major theme in the literature was that PYD programmes can promote positive development by instilling young people with an ability for intentional self-regulation. A final theme was how the development of intentional self-regulation and of positive assets protects young people from engagement in risk behaviours such as substance use and violence.

#### Intentional self-regulation

Busseri et al. [47], Schwartz et al. [56] and Lerner et al. [54] all suggest that PYD helps young people develop capacities for ‘intentional self-regulation’:

“*intentional self regulation may involve the selection of positive goals (e.g., choosing goals that reflect important life purposes), using cognitive and behavioral skills (such as executive functioning or resource recruitment) to optimize the chances of actualizing ones purposes and, when goals are blocked or when initial attempts at optimization fail, possessing the capacity to compensate effectively*”. (Lerner et al. [54] p.1108)

PYD can help adolescents reflect on existing behaviour, select personal goals and activities through which to pursue these, and apply available resources to pursue these goals and activities [42].

Lerner et al. [54] suggest that by promoting young people’s ability to intentionally self-regulate, PYD promotes positive interactions between individuals and their environments (or ‘developmental regulations’), to occur whereby young people are better able to capitalise on opportunities present within their environments in order to develop more and more positive assets of the sorts listed earlier (Lerner et al. [54]; Benson [42]; Busseri et al. [47]; Schwartz et al. [56]).

However, these authors are not explicit about how PYD interventions actually enable young people to develop their capacity for intentional self-regulation. For example, Benson [42] only offers generalities such as the suggestion that PYD does this by:

“*[i]ncreasing the developmental attentiveness of contexts…to increase their capacity to nurture, support, and constructively challenge the developing person, … [e]nhancing the skills and competencies of youth (to further enable their “natural” capacity to engage with, connect, change, and learn from their social contexts…[and [c]reating processes and opportunities to invite youth to actively exercise and utilize their capacity to engage with and change their social contexts*” (p.39).

#### How PYD might promote intentional self-regulation

This quote, as well as the theoretical literature more generally, stops short of explaining exactly how PYD programmes might promote better intentional self-regulation. However, informed by our earlier synthesis of PYD’s normative theory, we might fill in the gaps. First, PYD might provide individuals with the resources, in the form of relationships and training in specific skills which are the critical inputs upon which the use of intentional self-regulation within specific intervention-related activities depends. Second, PYD might enable individuals to practice intentional self-regulation in the context of multiple activities and settings so that they improve their general ability to intentionally self-regulate. Busseri et al. [47] come closest to making this explicit. They argue that PYD can provide a range of “*affordances*” (p.907) resources individuals use in the course of their development (e.g. relationships, challenges, education) to which young people may respond. Different individuals at different points in their maturation, with different needs and goals, will make use of different affordances. Third, PYD might refocus individuals’ existing capacities for intentional self-regulation away from anti-social goals and towards pro-social goals. This might occur via rewarding young people when they abandon anti-social activities and engage in specific pro-social activities. Kim et al. [51] for example refer to social learning theory to suggest that, by providing positive examples and celebrating achievements in the realm of pro-social activities, PYD programmes can reinforce positive behaviours and bonds to conventional society.

#### How PYD might reduce risk behaviours

The way in which PYD might reduce risk behaviours, such as substance use and violence, was a relatively minor theme in the literature, a limitation acknowledged by Kia-Keating et al. [50] and Lerner et al. [54]. The PYD literature offers a number of general suggestions about how the development of positive assets might reduce risks as well as a few isolated examples of how specific assets might protect against risk, but stops short of offering a comprehensive theory of change for how PYD reduces risk.

#### Buffering and compensation

Positive assets might reduce risk by processes of *‘buffering’* (Catalano et al. [48] p.233; Kia-Keating et al. [50]) or “compensation” (Busseri et al. [47] p.912). N.B. this use of the term ‘compensation’ differs from that cited above to describe one of the stages of intentional self-regulation.

Buffering is described as a process whereby risk factors in the environment have less influence on the behaviour of those with positive assets than those who lack these assets [48]. For example, individuals possessing the asset of a positive sense of identity might be less prone to peer pressure to engage in risk behaviours (Catalano et al. [48]; p.232).

Catalano et al. [48] describe ‘compensation’ in terms of those possessing positive assets being able to engage in risk with less harmful consequences. For example, a young person who is engaged with school might still participate in violence but with fewer harmful developmental effects. Schwartz et al. [56] refer to similar processes using slightly different terminology.

#### Pile-up and molecular impacts on risk

Pile-up is defined as occurring when the general accumulation of multiple assets regardless of their particular characteristics might lead to reduced risk behaviours. In contrast, molecular mechanisms occur when specific assets bring about reductions in risk behaviours because of their particular characteristics.

Other authors offer examples of assets which offer molecular protection against risk behaviors. Kim et al. [51] suggest that engagement with pro-social institutions will lead to reductions in anti-social behaviours. Benson and Scales [44] suggest that social skills, connections with pro-social peers and engagement with school will reduce involvement in substance use and violence via young people resolving conflict, modelling responsible behaviours and being reluctant to defy pro-social norms. Kia-Keating et al. [50] suggest that: social integration and self-efficacy will offer protection against conduct problems; pro-social behaviours will protect against substance use; emotional self-regulation will protect against externalising problem behaviours; adult supervision will protect against delinquency; and self-efficacy can interact with parental monitoring to protect against alcohol use. However these are piecemeal suggestions rather than a comprehensive theory of change.

## Discussion

### Summary of main results

Sixteen reports were included. All described PYD goals. All offered some insights into how PYD might reduce substance use and violence but none provided a comprehensive theory of change for how PYD might reduce substance use and violence. Different reports focused on different parts of the pathway by which PYD might prevent substance use and violence: some focusing on intentional self-regulation, some on the development of multiple, positive assets and some on how such assets might reduce risk behaviours. However, in doing so reports did not contradict each other and this enabled us to develop a more comprehensive theory of the overall pathway by which PYD might prevent substance use and violence, described below and summarised in Fig. 2.

Overall PYD interventions aim to provide young people with: positive expectations; enduring, affective relationships with adults; and diverse activities and settings. Participants learn ‘intentional self-regulation’, which involves: reflecting on existing behaviour; selecting personal goals and activities through which to pursue these; and using available resources to pursue these goals and activities. PYD interventions enable young people to learn and be rewarded for intentional self-regulation applied to multiple, mutually reinforcing intervention activities such as sports or arts. This then enables them to develop and apply intentional self-regulation more generally to other pro-social goals. As a result of developing intentional self-regulation, young people are better able to develop various positive assets (for example, the 5 C’s: competence, confidence, connection, character and caring). As these accrue, young people can make increasingly better use of the opportunities available in their wider environment. This enables further accrual of assets and ultimately young people contributing positively to their communities and societies, either maintaining or challenging existing arrangements.

These positive assets may then reduce risk behaviours via ‘buffering’, whereby environmental risk factors are less influential, or ‘compensation’, whereby young people still engage in risk behaviours but with fewer adverse consequences. Positive assets may reduce risk ‘molecularly’ (a specific asset offers protection against a specific risk), or via ‘pile-up’ (accumulation of multiple assets is generically protective). Molecular protection might occur for example when engagement with pro-social peer groups or institutions reduces anti-social behaviours, and when improved emotional self-regulation, social skills and self-efficacy enable better decision-making. The PYD theoretical literature however does not offer a comprehensive theory of change for which positive assets help prevent substance use or violence.

### Limitations

The main limitation of our review was in the assessment of quality. Unlike most theory syntheses, [35–37] we aimed to do this in order to give more weight in our synthesis to high-quality studies. Despite being informed by previous work [38, 57] and being accompanied by guidance, our criteria could not be applied consistently. If quality criteria are to be used in future syntheses of theoretical literature then more guidance is required on assessing these, particularly testability and parsimony.

There were also limitations in included reports. Although 16 reports is a good number for a review of theory, only nine of these provided a detailed consideration of how PYD might prevent risk behaviours. Much of the literature focused on asserting the normative value of PYD and consideration of causal theory was sometimes peripheral and unsystematic. Included reports failed to explain how PYD interventions aim to optimise young people’s capacity for intentional self-regulation or comprehensively explain how promoting positive assets could reduce risk behaviours. We nonetheless synthesised a theory of change by bringing together fragments from multiple reports. Our synthesis would have been less comprehensive had we only synthesised theoretical literature judged to be of high quality.

## Conclusions

This paper is an example of a rigorously conducted systematic synthesis of theoretical literature which builds on previous research by attempting to use quality assessment criterion and successfully applying qualitative synthesis to theoretical literature. By drawing together and filling gaps in the existing theoretical literature, we have developed a clear, comprehensive theory of change for how PYD interventions are intended to reduce substance use and violence. As mentioned in our introduction, our broader review also included a synthesis of empirical evaluations of current PYD interventions. Although not yet published, this element of the review found that interventions were not informed by the theories of change synthesised in this paper and so provided little useful evidence about the effectiveness of the PYD approach. The very fact that previous evaluations have not focused on theoretically informed interventions is evidence for the need for the present paper. We recommend that our theory of change be used in future systematic reviews to inform inclusion criteria. We also recommend that new empirical evaluations of the effectiveness of PYD draw on our theory of change when developing intervention logic models [58] to ensure that the interventions evaluated properly embody the PYD approach. We also hope our clear exposition of the PYD theory of change will help policy-makers and practitioners make more informed decisions about whether PYD interventions might be appropriate to local needs.

However, gaps remain, which PYD theorists should address, particularly regarding the pathways via which PYD programmes brings about intentional self-regulation and how particular assets might offer protection against particular risk behaviours. There is also a need for future theory syntheses to develop more useable criteria for assessing quality.

## Additional files

Additional file 1: **Search on Psycinfo (EBSCO) 7/11/2013.** (DOCX 15 kb)

Additional file 2: **PRISMA checklist.** (DOCX 22 kb)
