# Supplementary material for: What is positive youth development and how might it reduce substance use and violence? A systematic review and synthesis of theoretical literature
Source: BMC Public Health. 2016 Feb 10;16:135. doi: 10.1186/s12889-016-2817-3 (PMC4748512; doi:10.1186/s12889-016-2817-3)
Supplement: Supplementary file 1 — Search on Psycinfo (EBSCO) 7/11/2013. (DOCX 15 kb) [file 12889_2016_2817_MOESM1_ESM.docx]

**Web appendix 1: Search on Psycinfo (EBSCO) 7/11/2013**

Searcher: CS

Search modes - Boolean/Phrase

No. of records: 8227

*Notes:*

# = wildcard of 1 or 0 characters

* = truncation

N1 = words within 1 place of each other in any order

| **#** | **Query** | **Results** |
| --- | --- | --- |
| S1 | TI ( "Young people#" OR "young person#" OR "young offender#" OR adolescent# OR adolescence OR youth# OR minors OR teen OR teens OR teenage OR teenaged OR teenager# OR juvenile# OR pupil# OR boy# OR girl# OR underage# OR (school AND dropout#) OR (school AND "drop out#") OR "school aged") OR AB ( "Young people#" OR "young person#" OR "young offender#" OR adolescent# OR adolescence OR youth# OR minors OR teen OR teens OR teenage OR teenaged OR teenager# OR juvenile# OR pupil# OR boy# OR girl# OR underage# OR (school AND dropout#) OR (school AND "drop out#") OR "school aged") | 311,506 |
| S2 | TI "youth opportunit*" OR AB "youth opportunit*" | 36 |
| S3 | TI "youth work*" OR AB "youth work*" | 413 |
| S4 | TI "youth club#" OR AB "youth club#" | 74 |
| S5 | TI "youth centre#" OR AB "youth centre#" | 38 |
| S6 | TI "youth center#" OR AB "youth center#" | 145 |
| S7 | TI (youth# N2 empower*) OR AB (youth# N2 empower*) | 185 |
| S8 | TI (adolescent# N2 empower*) OR AB (adolescent# N2 empower*) | 112 |
| S9 | TI ("young people#" N3 empower*) OR AB ("young people#" N3 empower*) | 54 |
| S10 | TI ("youth led") OR AB ("youth led") | 51 |
| S11 | TI youth N1 voice# OR AB youth N1 voice# | 85 |
| S12 | TI youth N1 advoca* OR AB youth N1 advoca* | 78 |
| S13 | TI (youth# N1 engagement) OR TI ("engaging youth#") OR AB (youth# N1 engagement) OR AB("engaging youth#") | 249 |
| S14 | TI (adolescent# N1 engagement) OR TI ("engaging adolescent#") OR AB (adolescent# N1 engagement) OR AB ("engaging adolescent#") | 286 |
| S15 | TI ( "young people#" N1 (engagement OR engaging) ) OR AB ( "young people#" N1 (engagement OR engaging) ) | 92 |
| S16 | TI (youth# N1 involvement) OR TI ("involving youth#") OR AB (youth# N1 involvement) OR AB ("involving youth#") | 402 |
| S17 | TI ( "young people#" N1 (involving OR involvement) ) OR AB ( "young people#" N1 (involving OR involvement) ) | 111 |
| S18 | TI "youth participation" OR AB "youth participation" | 191 |
| S19 | TI "adolescent participation" OR AB "adolescent participation" | 85 |
| S21 | TI "youth program*" OR AB "youth program*" | 306 |
| S22 | TI youth N5 asset# OR AB youth N5 asset# | 178 |
| S23 | TI youth N12 asset# OR AB youth N12 asset# | 245 |
| S24 | TI "young people#" N12 asset# OR AB "young people#" N12 asset# | 32 |
| S25 | TI adolescent# N12 asset# OR AB adolescent# N12 asset# | 153 |
| S26 | AB (("positive development" ) N12 ("young people#" OR adolescen* OR youth# OR minors OR teen* OR juvenile* OR pupil* OR boy# OR girl# OR "school aged" OR "drop out#" OR dropout# OR underage#) ) | 174 |
| S27 | TI "extra curricular" OR AB "extra curricular" | 419 |
| S28 | TI supplement* N1 education OR AB supplement* N1 education | 98 |
| S29 | TI (nonformal* N1 education) OR AB (nonformal* N1 education) OR TI ("non formal*" N1 education) OR AB ("non formal*" N1 education) | 92 |
| S30 | TI informal* N1 education OR AB informal* N1 education | 281 |
| S31 | TI "additional education" OR AB "additional education" | 144 |
| S32 | TI (community N1 empower*) OR AB (community N1 empower*) | 512 |
| S33 | TI "civic engagement" OR AB "civic engagement" | 744 |
| S34 | TI "Asset# building" OR AB "Asset# building" | 87 |
| S35 | TI "Developmental asset#" OR AB "Developmental asset#" | 172 |
| S36 | TI "Psychosocial asset#" OR AB "Psychosocial asset#" | 24 |
| S37 | TI "peer led" OR AB "peer led" | 414 |
| S38 | TI "peer engagement" OR AB "peer engagement" | 15 |
| S39 | TI service N1 learning OR AB service N1 learning | 1,601 |
| S40 | TI afterschool OR AB afterschool | 184 |
| S41 | TI ( "after school" N5 (program# OR club# OR initiative# OR scheme# OR center# OR centre# OR event# OR intervention# OR service# OR project# OR service# OR session#) ) OR AB ( "after school" N5 (program# OR club# OR initiative# OR scheme# OR center# OR centre# OR event# OR intervention# OR service# OR project# OR service# OR session#) ) | 957 |
| S42 | S27 OR S28 OR S29 OR S30 OR S31 OR S32 OR S33 OR S34 OR S35 OR S36 OR S37 OR S38 OR S39 OR S40 OR S41 | 5,553 |
| S43 | TI ( mentor* N12 ("young people#" OR adolescen* OR youth# OR minors OR teen* OR juvenile* OR pupil* OR boy# OR girl# OR "school aged" OR "drop out#" OR dropout# OR underage#) ) OR AB ( mentor* N12 ("young people#" OR adolescen* OR youth# OR minors OR teen* OR juvenile* OR pupil* OR boy# OR girl# OR "school aged" OR "drop out#" OR dropout# OR underage#) ) | 826 |
| S44 | TI ( coach* N12 ("young people#" OR adolescen* OR youth# OR minors OR teen* OR juvenile* OR pupil* OR boy# OR girl# OR "school aged" OR "drop out#" OR dropout# OR underage#) ) OR AB ( coach* N12 ("young people#" OR adolescen* OR youth# OR minors OR teen* OR juvenile* OR pupil* OR boy# OR girl# OR "school aged" OR "drop out#" OR dropout# OR underage#) ) | 523 |
| S45 | TI ( "development program*" N12 ("young people#" OR adolescen* OR youth# OR minors OR teen* OR juvenile* OR pupil* OR boy# OR girl# OR "school aged" OR "drop out#" OR dropout# OR underage#) ) OR AB ( "development program*" N12 ("young people#" OR adolescen* OR youth# OR minors OR teen* OR juvenile* OR pupil* OR boy# OR girl# OR "school aged" OR "drop out#" OR dropout# OR underage#) ) | 344 |
| S46 | TI ( (community N1 service) N12 ("young people#" OR adolescen* OR youth# OR minors OR teen* OR juvenile* OR pupil* OR boy# OR girl# OR "school aged" OR "drop out#" OR dropout# OR underage#) ) OR AB ( (community N1 service) N12 ("young people#" OR adolescen* OR youth# OR minors OR teen* OR juvenile* OR pupil* OR boy# OR girl# OR "school aged" OR "drop out#" OR dropout# OR underage#) ) | 327 |
| S47 | DE "Social Programs" OR DE "Mentor" OR DE "Coaching" | 6,995 |
| S48 | TI "positive youth" OR AB "positive youth" | 810 |
| S49 | TI "youth development" OR AB "youth development" | 1,750 |
| S50 | S2 OR S3 OR S4 OR S5 OR S6 OR S7 OR S8 OR S9 OR S10 OR S11 OR S12 OR S13 OR S14 OR S15 OR S16 OR S17 OR S18 OR S19 OR S20 OR S21 OR S22 OR S23 OR S24 OR S25 OR S26 OR S43 OR S44 OR S45 OR S46 OR S48 OR S49 | 6,245 |
| S51 | S42 OR S47 | 12,467 |
| S52 | S51 AND S1 | 2,564 |
| S53 | S50 OR S52  Limiters - Publication Year: 1985-2013 Search modes - Boolean/Phrase | 7,286 |
| S54 | S51  Limiters - Publication Year: 1985-2013; Age Groups: School Age (6-12 yrs), Adolescence (13-17 yrs) | 2,516 |
| S55 | S54 OR S53 | 8,227 |
